# Development of a robust radiomic biomarker of progression-free survival in advanced non-small cell lung cancer patients treated with first-line immunotherapy

**DOI:** 10.1038/s41598-022-14160-7

**Published:** 2022-06-15

**Authors:** Apurva Singh, Hannah Horng, Leonid Roshkovan, Joanna K. Weeks, Michelle Hershman, Peter Noël, José Marcio Luna, Eric A. Cohen, Lauren Pantalone, Russell T. Shinohara, Joshua M. Bauml, Jeffrey C. Thompson, Charu Aggarwal, Erica L. Carpenter, Sharyn I. Katz, Despina Kontos

**Affiliations:** 1 Department of Radiology, Center for Biomedical Image Computing and Analytics (CBICA), University of Pennsylvania, Rm D702 Richards Bldg., 3700 Hamilton Walk, Philadelphia, PA 19104 USA; 2 Department of Bioengineering, University of Pennsylvania, Philadelphia, PA 19104 USA; 3 Department of Biostatistics, Epidemiology, and Informatics, University of Pennsylvania, Philadelphia, PA 19104 USA; 4 Department of Medicine, Division of Hematology-Oncology, University of Pennsylvania, Philadelphia, PA 19104 USA; 5 Department of Medicine, Pulmonary, Allergy and Critical Care Medicine, Thoracic Oncology Group, University of Pennsylvania, Philadelphia, PA 19104 USA

**Keywords:** Cancer, Computational biology and bioinformatics, Biomarkers

## Abstract

We aim to determine the feasibility of a novel radiomic biomarker that can integrate with other established clinical prognostic factors to predict progression-free survival (PFS) in patients with non-small cell lung cancer (NSCLC) undergoing first-line immunotherapy. Our study includes 107 patients with stage 4 NSCLC treated with pembrolizumab-based therapy (monotherapy: 30%, combination chemotherapy: 70%). The ITK-SNAP software was used for 3D tumor volume segmentation from pre-therapy CT scans. Radiomic features (n = 102) were extracted using the CaPTk software. Impact of heterogeneity introduced by image physical dimensions (voxel spacing parameters) and acquisition parameters (contrast enhancement and CT reconstruction kernel) was mitigated by resampling the images to the minimum voxel spacing parameters and harmonization by a nested ComBat technique. This technique was initialized with radiomic features, clinical factors of age, sex, race, PD-L1 expression, ECOG status, body mass index (BMI), smoking status, recurrence event and months of progression-free survival, and image acquisition parameters as batch variables. Two phenotypes were identified using unsupervised hierarchical clustering of harmonized features. Prognostic factors, including PDL1 expression, ECOG status, BMI and smoking status, were combined with radiomic phenotypes in Cox regression models of PFS and Kaplan Meier (KM) curve-fitting. Cox model based on clinical factors had a c-statistic of 0.57, which increased to 0.63 upon addition of phenotypes derived from harmonized features. There were statistically significant differences in survival outcomes stratified by clinical covariates, as measured by the log-rank test (*p* = 0.034), which improved upon addition of phenotypes (*p* = 0.00022). We found that mitigation of heterogeneity by image resampling and nested ComBat harmonization improves prognostic value of phenotypes, resulting in better prediction of PFS when added to other prognostic variables.

## Introduction

The overexpression of programmed cell death-ligand 1 (PD-L1) on the surface of tumor cells allows them to escape T cell mediated apoptosis, due to the interaction of PD-L1 with programmed cell death protein 1 (PD-1)^1^. Recognition of this mechanism of immune escape by tumors has led to the rise of immune checkpoint inhibitor therapy to treat a variety of advanced malignancies. Pembrolizumab (PEMBRO) is one such immune checkpoint inhibitor and is a humanized monoclonal antibody directed to PD-1, resulting in the disruption of the PD1/PDL1 axis. PEMBRO is now an approved first-line therapy for NSCLC^2^, however, determining which patients may benefit from PEMBRO remains a challenge. Tumor expression of PD-L1, the currently accepted predictive biomarker for use of PEMBRO, is an imperfect predictor of therapy response. PDL1 enriched tumors receiving anti-PD1/PDL1 monotherapy exhibit a 40–50% response to therapy and with the addition of chemotherapy this response rate can increase to 60–70% in NSCLC patients receiving 1st line therapy^3^. Nevertheless, up to 30–40% of NSCLC patients with high PDL1 expression exhibit primary resistance to therapy, and tumors with very low PDL1 expression can respond even without additional chemotherapy^4^. Additionally, PD-L1 demonstrates intra-tumoral heterogeneity and the expression levels are dynamic over time^5,6^. Thus tumor expression level of PDL1 alone is inadequate as a biomarker to predict response to anti-PD1/PDL1 therapy. For this reason, there is a critical need to identify additional predictive biomarkers to better determine the subgroup of patients mostly likely to benefit from therapy with these agents. This is important because anti-PD1/PDL1 agents carry significant toxicity, are expensive and switching to a potentially effective therapy is delayed which may translate into lowered overall survival.

The field of radiomics enables the detection of various patterns in standard-of-care medical images not discernible by the human eye^7,8^. Radiomics can provide longitudinal, in-depth analysis of heterogeneity of the entire tumor and surrounding tissues in vivo^9,10^*.* By comparison, standard biopsy procedures are susceptible to sampling error and come at a significant cost of patient discomfort, iatrogenic complications and financial cost to the health-care system^11,12^ and as such are typically limited to use at initial diagnosis and to document recurrence. Preliminary studies in the field of immunotherapy suggest that radiomic signatures exist that correlate with anti-PDL1/PD1 therapy response. For example, a study by Trebeschi et al*.* identified predictive features in pre-treatment CT imaging for patients undergoing immunotherapy for melanoma (n = 203 patients) and NSCLC (n = 123 patients) in a non-1st line therapy cohort. They developed a machine learning biomarker from radiomic descriptors of tumor morphology, capable of distinguishing between immunotherapy responders and non-responders^13^. Another study by Sun et al*.* examined radiomic features in baseline CT imaging of patients with solid tumors first in a training set (n = 135 patients) then in 3 validation cohorts (n = 100–137 patients) all receiving anti-PD-1 or anti-PD-L1 monotherapy. From these images they developed a radiomics-based biomarker of tumor infiltration with CD8 cells that correlated with patient outcomes^14^. A recent study by Vaidya et al*.* in advanced NSCLC (n = 109 patients) undergoing anti-PDL1/PD1 monotherapy used 198 radiomic textural patterns extracted from within and around the target nodules on the baseline CT images. The radiomic model built using these features was able to identify patients at high-risk for hyper progression^15^. Zerunian et al. aimed to assess whether CT-derived texture parameters can predict overall survival (OS) and progression-free survival (PFS) in 21 patients with advanced NSCLC who had been treated with first-line pembrolizumab. The Kaplan Meier analysis showed that MPP (mean value of positive pixels) texture features were significantly associated with lower OS and PFS^16^.

While these preliminary studies highlight a potential role for radiomics as an imaging biomarker in the management of NSCLC receiving immunotherapy, they also have important limitations. One limitation is the incorporation of radiomic information in the form of single-feature predictors. Radiomic feature sets are usually large in number, and this approach can lead to errors in the identification of significant features and limit identification of phenotypic patterns arising from combinations of features (i.e., signatures). In addition, these studies did not examine the added value of radiomics features to established clinical prognostic factors including PDL1 expression. Another limitation is that these studies was the inherent variability of the imaging data including the use of patients who have received prior lines of therapy, which would be anticipated to have therapy-induced altered tumor microenvironment, and heterogeneous imaging protocols. The latter is important since radiomic features are influenced by image acquisition (type of scanner, patient motion, use of contrast, reconstruction kernel, field-of-view, slice thickness, etc.) and post-processing techniques. For example, in a study performed by Zhao et al. on a cohort of same-day repeated CT scans demonstrated that interchanging smooth and sharp reconstruction algorithms can reduce feature reproducibility^17^. In another study, CT scans were obtained at different dose levels, section thicknesses, kernels, and reconstruction algorithm settings, only intensity, shape, and texture radiomic features were found to be reproducible across settings^18^. Prior preliminary immunotherapy studies did not explore the effect of heterogeneity in imaging parameters on the robustness of the radiomic features.

In our study, we develop a novel radiomic biomarker that integrates with PDL1 expression, ECOG status, BMI, and smoking status data to enhance precision in progression-free survival prediction in 107 patients with stage 4 NSCLC treated at our institution with first-line PEMBRO monotherapy or combination therapy. We evaluate a novel phenotyping approach based on unsupervised hierarchical clustering, which can effectively reduce the number of radiomic predictors while retaining important information in identifying statistically significant phenotypic signatures composed of multiple rather than individual features. We demonstrate the efficacy of the Cancer Phenomics Toolkit (CapTk)^19^, a highly-standardized, user-friendly, open-source software developed at our institution, that conforms to the Imaging Biomarker Standardisation Initiative (IBSI) radiomics standardization protocols^20^, for radiomics analysis. We account for differences in image parameters by resampling the images to the minimum voxel spacing parameters and using a nested ComBat approach for harmonization^21^ by the image acquisition parameters. ComBat is a harmonization method originally developed for genomics that can correct variation in features due to imaging parameters by using empirical Bayes to estimate location and scale parameters to shift data. In previous studies, ComBat has been shown to harmonize radiomic features from different CT protocols as well as reduce percentage of features with significantly different distributions by batch effect^22,23^. While ComBat is fast and easy to use, current implementations of ComBat are only able to harmonize by a single batch effect at a time and are therefore unable to adequately harmonize datasets that are heterogeneous in more than one batch effect. The nested ComBat approach used in our study enables harmonization by multiple batch effects by implementing sequential harmonization. We hypothesize that the addition of the radiomic phenotypes to established clinical biomarkers including PDL1, will improve the accuracy of the prognostic model in the prediction of progression-free survival in the patients with NSCLC. We also hypothesize that mitigation of the heterogeneity introduced by the voxel spacing and image acquisition parameters in radiomic features will improve the prognostic performance of the radiomic phenotypes.

## Materials and methods

### Study sample and data

This single-centre retrospective, observational study was conducted at the Hospital of the University of Pennsylvania between November 2016 to December 2020. The study was approved by the University of Pennsylvania’s Institutional Review Board (IRB) committee (IRB protocol number—848796) under a waiver of informed consent. All methods in this study were in accordance with the Declaration of Helsinki. Patients with stage 4 NSCLC treated with first-line pembrolizumab based therapy at our institution were identified. The details of the monotherapy and combination therapy regimens are included in Supplementary Table S1. The shooting ranges of the CT scans were the thoracic regions of the patients. The 3D tumor CT volumes were manually segmented by board-certified, fellowship-trained thoracic radiologists S.I.K (with 18 years of clinical experience) and L.R. (with 4 years of clinical experience) using the semi-automated ITK-SNAP software (version 3.6.0)^20^.

### Radiomic feature extraction

The impact of heterogeneity introduced by differences in the physical dimensions of the images was mitigated by performing anisotropic resampling. The images were resampled to the minimum value across each of the voxel spacing parameters (x*y*z − 0.54 mm, 0.54 mm, 0.8 mm). A total of 102 radiomic features were extracted from each tumor’s entire segmented volume in the resampled images, using CaPTk^19^. The CaPTk software can visualize the overall tumor burden of lung cancer, including metastatic lesions. However, in our study, only primary lesions were analyzed. The radiomic features represent the following eight type of descriptors: (1) *Intensity features or first-order statistics*—These features capture the voxel grey-level intensities within a neighborhood. (2) *Histogram-based features*—The computation of these features involves the generation of an intensity histogram by discretization of the original intensity distribution. (3) *Volumetric features*—These features are computed by utilizing the voxel intensities in the ROI and are based on the relationship between discretized intensity and the fraction of the volume containing the least intensity. (4) *Morphologic features*—These features describe geometric aspects of a region of interest (ROI), such as area and volume. (5) *Gray level run length matrix features*—These features are based on quantifying gray level runs as the lengths of consecutive pixels. (6) *Neighboring gray tone difference matrix features*—They are rotation-independent features based on gray-level relationships between neighboring voxels and aim to capture the coarseness of the overall texture. (7) *Gray level size zone matrix features*—The grey level size zone matrix (GLSZM) counts the number of groups (or zones) of linked voxels. Voxels are linked if the neighboring voxel has an identical discretized grey level. Whether a voxel classifies as a neighbor depends on its connectedness. (8) *Local binary pattern features (LBP)*—These features are used to describe the local texture patterns in an image. The LBP works in a block size of 3 × 3, in which the center pixel is used as a threshold for the neighboring pixel, and the LBP code of a center pixel is generated by encoding the computed threshold value into a decimal value. A list of features belonging to each family which were used in the analysis and their formulae can be found in Supplementary Tables S4 and S5 respectively.

### Radiomic feature harmonization

The impact of heterogeneity introduced by the image acquisition parameters (Table 1) was mitigated using a nested ComBat approach. The original ComBat method was developed to harmonize by a single covariate^21^, so we incorporated a “nested” approach to correct for multiple batch effects. The nested approach was initialized with the original radiomic features as the input data and a list of batch effects—in this case, contrast enhancement and kernel resolution, resulting in two batch effects. Age, sex, race, PD-L1 expression, ECOG status, body mass index (BMI), smoking status, recurrence event and months of progression-free survival were all protected during harmonization to prevent removal of biological variation of interest. Harmonization order was determined by iterating through all possible permutations of the list of two batch effects. At each iteration, the original input data was sequentially harmonized with ComBat where the original data was harmonized by the first batch effect in the permutation corresponding to the iteration, then the resulting harmonized data was harmonized again by the second batch effect in the permutation, resulting in a feature set that had been harmonized two times. The resulting harmonized feature sets were each assessed for significant differences between distributions for each batch effect using the Anderson–Darling (AD) test^24^ at a *p* value significance level of 0.05. The harmonized feature set with the lowest number of features with detected differences in distribution across all batch effects was selected as the final output. Features remaining significantly affected by batch effects after ComBat harmonization were deemed as non-robust and discarded from further analysis.

**Table 1:** Batch effects introduced by image acquisition parameters.

Batch effect	Category	Number of patients (n = 107)
Contrast enhancement	Contrast-enhanced	80 (74.8%)
	Non-contrast-enhanced	27 (25.2%)
Kernel resolution	Low resolution-soft tissue kernel (≤ B40f (Siemens), B, C, D (Philips), STD (GE)	90 (84.1%)
	High resolution-lung kernel > B40f (Siemens), A (Philips), LUNG (GE)	17 (15.8%)

### Radiomic phenotype identification

To identify intrinsic imaging phenotypes, unsupervised hierarchical clustering^25^ was performed on the harmonized and reduced feature set. The input to the clustering algorithm is a set of feature vectors, consisting of the harmonized features for each scan. The number of distinct clusters *k* obtained from the unsupervised hierarchical clustering algorithm are interpreted as intrinsic imaging phenotypes in the cohort. An agglomerative approach was used to create a hierarchical clustering of the patients using Euclidean distance for distance between the feature vectors and Ward’s minimum variance method as the clustering criterion^26^. The optimal number of distinct phenotypes, k, was determined by assessing the stability and significance of each phenotype for each value of k that was considered. The optimal number of stable phenotypes was determined using consensus clustering^27^, where dataset was sub-sampled and cluster arrangements were determined using varying values of k. For each value of k, the proportion that two patients occupied the same phenotype cluster out of the number of times they appeared in the same subsample was determined and stored in a consensus matrix, from which a cumulative distribution function (CDF) was determined. Cluster stability, determined by the area under the CDF curve, was evaluated for each value of k. Statistical significance of the identified, stable phenotypes was evaluated using the SigClust method^28,29^. Here, the significance of the cluster index, defined as the sum of within-cluster sums of squares about the cluster-mean divided by the total sum of squares about the overall mean was tested against a null distribution, simulated using 10,000 samples from a Gaussian distribution fit to the data. The test was performed at each phenotype split to determine statistical significance (*p* < 0.05).

### Details of prognostic models and their association with survival outcome

Kaplan–Meier (K–M) curves and log-rank test using the *entire* cohort assessed the performance of the model built using the radiomic phenotypes derived from the harmonized feature set (where radiomic phenotype cluster was coded as a categorical variable, thus reducing the dimensionality of the original feature set) in separating participants above versus below the median score during the prognosis of progression-free survival (PFS)^30^. The *p* value from the log-rank test indicates if the model can provide a statistically significant (*p* < 0.05) separation between patients with prognostic scores above and below the median prognostic score. The performance of this model is compared with that of models built using only the clinical covariates (PD-L1 expression, ECOG, BMI and smoking status (Table 2)) and with a multivariable model built with a combination of the clinical covariates and the radiomic phenotypes derived from the harmonized features, to measure the improvement in prognostic ability caused by the combination of radiomic and established clinical prognostic factors. The performance of the model using the radiomic phenotypes derived from the harmonized radiomic features was also compared with the performance of the model built using the radiomic phenotype derived from the non-harmonized features, to evaluate the impact of harmonization on the prognostic ability of the features.

**Table 2:** Clinical covariate categories and number of patients.

Clinical covariate	Category	Number of patients (n = 107)
PDL1 expression	PDL1 < 10%	52 (48.6%)
	10% ≤ PDL1 < 50%	16 (15%)
	PDL1 ≥ 50%	39 (36.4%)
BMI	Underweight (BMI < 18.5)	2 (1.8%)
	Normal (18.5 ≤ BMI ≤ 24.9)	37 (34.6%)
	Overweight (25 ≤ BMI ≤ 29.9)	39 (36.4%)
	Obese (BMI ≥ 30)	29 (27.1%)
Smoking status	Former smoker	54 (50.5%)
	Current smoker	39 (36.4%)
	Non-smoker	14 (13.1%)
ECOG performance status	Value 0	35 (32.7%)
	Value 1	52 (48.6%)
	Value 2	15 (14.0%)
	Value 3	5 (4.7%)

To analyze the difference in survival outcomes between patients treated with PEMBRO monotherapy vs. combination therapy (PEMBRO + chemotherapy), we also performed stratified analysis to evaluate K–M curves using the log-rank test to assess the performance of the prognostic models for patients who received monotherapy compared to patients who received combination therapy.

Further, fivefold cross-validated Cox proportional-hazards regression analysis with 200 iterations was performed on all the models described above. The discrimination capacity of the models was assessed using the concordance statistic (c-statistic)^31^.

### Assessment of the biological significance of the radiomic phenotypes

The biological significance of the radiomic phenotypes was studied by assessing the ability of the phenotypes to predict PD-L1 expression of the tumors using a random forest classifier. The predictor and outcome variables were divided into training and test sets (70:30 ratio) and fivefold cross-validated AUCs for the prediction of PDL1 expression were computed. The distribution of the continuous clinical variables of PDL1 expression and BMI across patients belonging to the two phenotypes was also compared using boxplots. The relation between the radiomic phenotypes and the categorical clinical variables (PDL1 expression, BMI, ECOG and smoking status) was tested using the chi-square test, with the null hypothesis that the clinical variables are not associated with the phenotypes.

We performed all data manipulation, statistical analysis, and plotting using Python (Ver. 3.7, Anaconda) and the R programming language (Ver. 3.5.1)^32,33^.

## Results

### Patient characteristics

The median age of the patients was 67 years (range [38, 90] years), 49% of the cohort was male (Table 3) and 36% of the cohort consisted of current smokers (Table 2). The entire cohort received first-line therapy with PEMBRO, with 28.9% receiving monotherapy (PEMBRO) and 71.1% receiving combination therapy (Table 4). The details of the therapy are included in Supplementary Table S1.

**Table 3:** Patient demographic information.

Demographic variable	Category	Number of patients (n = 107)
Sex	Male	52 (48.6%)
	Female	55 (51.4%)
Race	White	73 (68.2%)
	Black or African American	29 (27.1%)
	Latino	1 (0.9%)
	Asian	1 (0.9%)
	Other	3 (2.8%)
Age (years)	Median, range	67, [38, 90]

**Table 4:** Type of therapy administered to the cohort.

Type of therapy	Number of patients (n = 107)
Monotherapy	31 (28.9%)
Combination therapy	4 (3.7%)
	2 (1.8%)
	2 (1.8%)
	1 (0.9%)
	67 (62.6%)

### Radiomic feature harmonization

The percentage of features with significantly different distributions arising from the batch effects was reduced after applying nested ComBat to the original features (Supplementary Table S2). A total of 64.7% of the original radiomic features were robust to differences introduced by the batch effects.

### Radiomic phenotype identification

**Figure 1:**
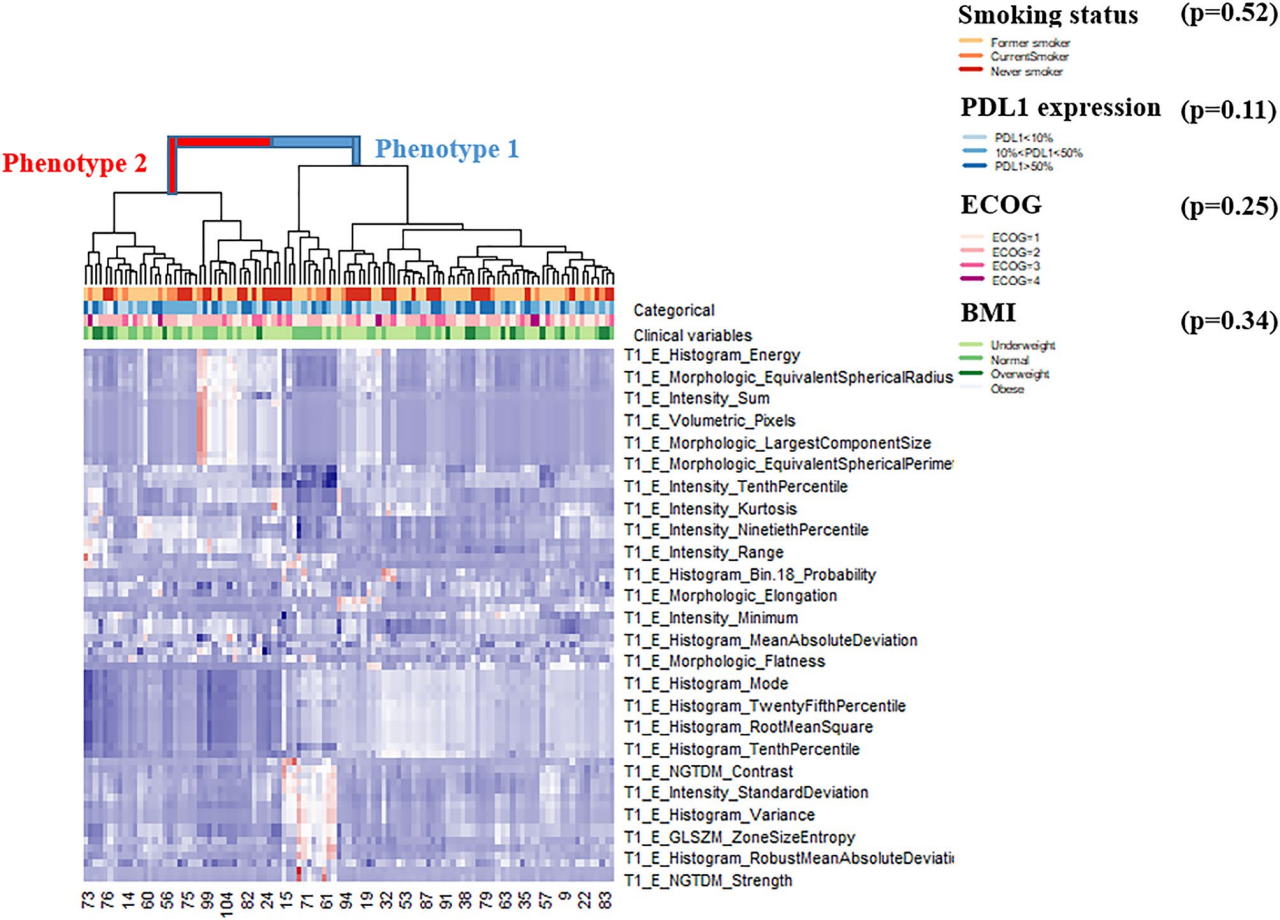
Heatmap of radiomic derived features (created using R programming language (ver. 3.5.1) https://www.R-project.org/). Unsupervised hierarchical clustering identifies two distinct, and statistically significant (*p* = 0.02) tumor radiomic phenotypes. Association of these phenotypes with the clinical covariates is assessed by the Chi square test and the resultant *p* values are included in the figure.

From the 102 initially extracted radiomic features, 34.6% of the features were significantly affected by batch effects after ComBat harmonization and were dropped. Two distinct radiomic phenotypes were identified, with 56 tumors in phenotype 1 and 51 tumors in phenotype 2 (Fig. 1) (*p* = 0.02 for SigClust test of two clusters versus one). The cumulative density function curves generated from the harmonized and non-harmonized feature sets are included in the Supplementary Fig. S1. The radiomic phenotypes derived from the 102 non-harmonized feature set are included in Supplementary Fig. S3.

### Radiomic phenotype association with outcomes

**Figure 2:**
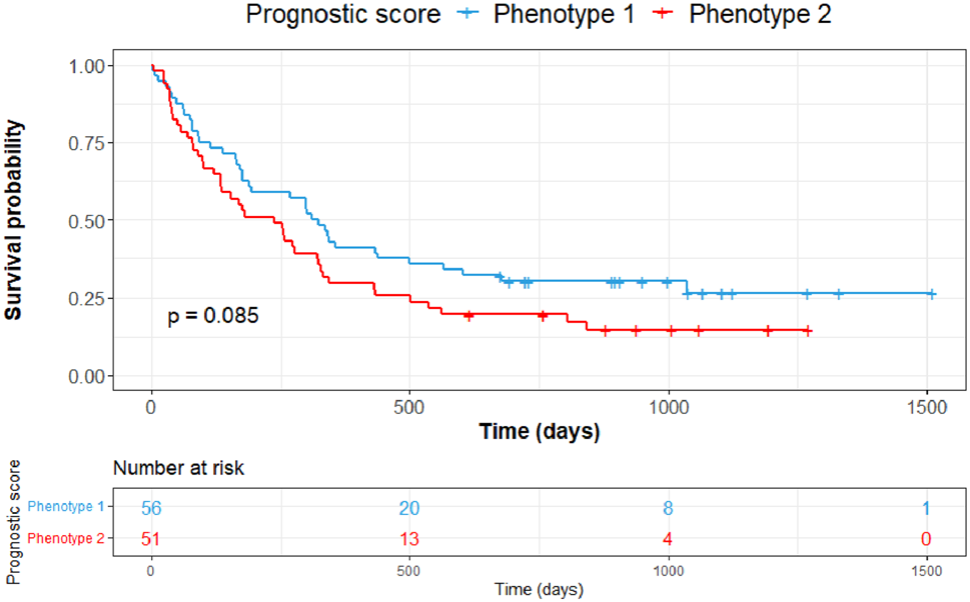
Survival analysis by radiomic phenotypes generated from harmonized radiomic features.

**Figure 3:**
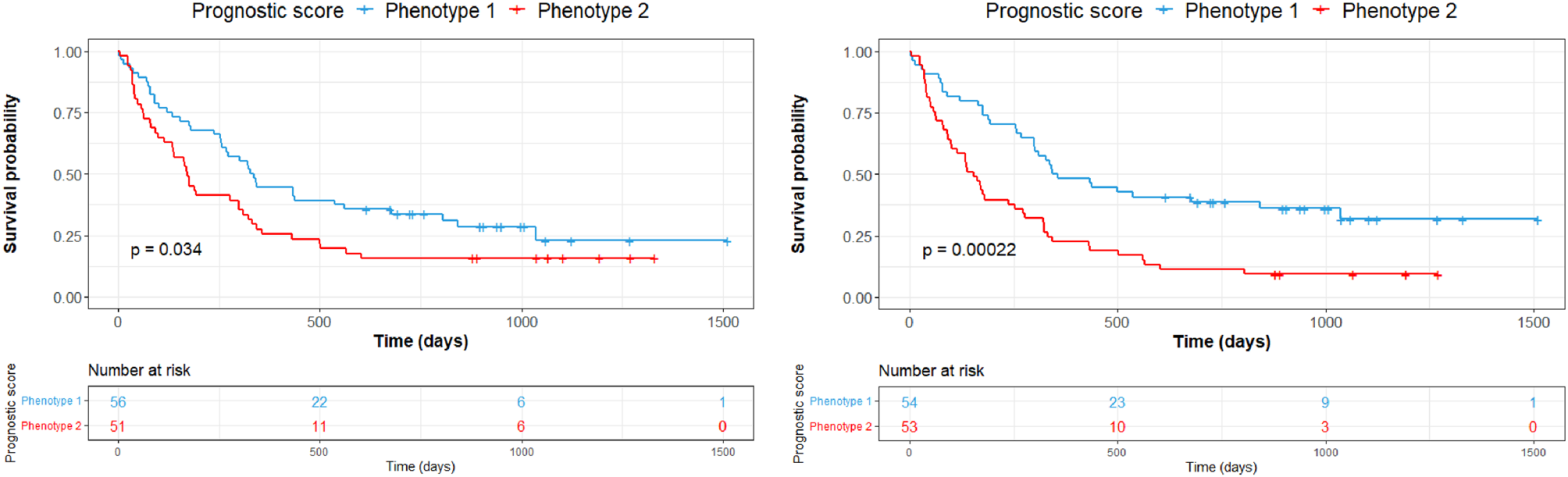
Survival analysis using multivariable models. Progression-free survival for the multivariable model built using only clinical covariates (PDL1 expression, ECOG, BMI and smoking status) (left) and for the multivariable model built using clinical covariates and radiomic phenotypes.

**Figure 4:**
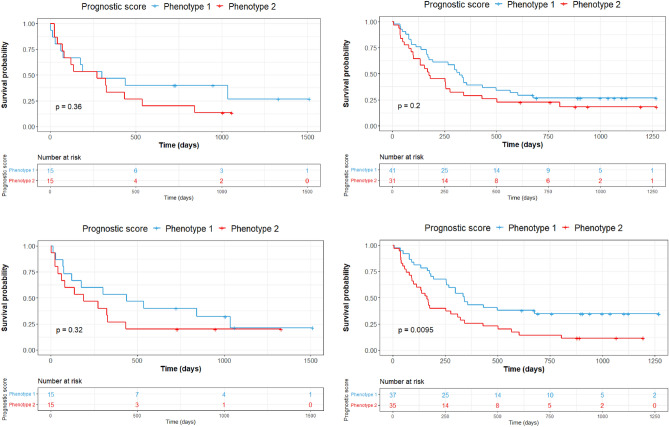
Survival analysis by line of therapy. Progression-free survival for the model built using radiomic phenotypes of patients treated with monotherapy (top left), the model built using radiomic phenotypes of patients treated with combination therapy (top right), multivariable model built using clinical covariates (PDL1 expression, ECOG, BMI and smoking status) and radiomic phenotypes of patients treated with monotherapy (bottom left) and multivariable model built using clinical covariates and radiomic phenotypes of patients treated with combination therapy (bottom right).

Median PFS was 270 days (8.8 months) for patients belonging to phenotype 1 versus 273 days (8.9 months) for those belonging to phenotype 2. The split between K–M curves for PFS resulted in log-rank *p* = 0.085 for tumors with radiomic phenotype 2 versus 1 (Fig. 2). The multivariable model of PFS, incorporating clinical covariates (PDL1 expression, ECOG status, BMI and smoking status), yielded log-rank *p* = 0.034 with a statistically significant separation of K–M curves for patients above versus below the median prognostic score. The multivariable model of PFS incorporating radiomic phenotypes with clinical covariates increased the separation of the curves and yielded log-rank *p* = 0.00022 (Fig. 3). When PFS was analyzed by line of therapy using the multivariable model involving phenotypes and clinical variables, a statistically significant separation of the K–M curves for patients above versus below the median prognostic score was observed for patients treated with combination therapy (log-rank *p* = 0.0095), whereas there was no appreciable separation for patients who received monotherapy (log-rank *p* = 0.32) (Fig. 4). We also built a multivariable model combining the radiomic phenotypes derived from the original feature set without ComBat harmonization, with the clinical covariates. No significant separation of the K–M curves was observed for patients above versus below the median prognostic score (log-rank *p* = 0.16) for all models (Supplementary Figs. S3 and S4).

The PFS model with the tumor volume (cm^3^) yielded a c-statistic of 0.47 (95% CI 0.45–0.52); the PFS model with the radiomic phenotypes yielded a c-statistic of 0.55 (95% CI 0.52–0.61); a multivariable model using only clinical covariates gave a c-statistic of 0.57 (95% CI 0.53–0.62); including tumor volume with the clinical covariates gave a c-statistic of 0.58 (95% CI 0.52–0.60); and including radiomic phenotypes with the clinical covariates in the multivariable model increased the c-statistic to 0.63 (95% CI 0.54–0.64) (Table 5). A multivariable model built from the original features without ComBat harmonization yielded a c-statistic of 0.51 (95% CI 0.49–0.54) (Supplementary Table S3).

**Table 5:** Progression-free survival Cox proportional-hazards regression analysis c-scores.

Model	Components	Fivefold cross-validated c-score, 95% CI
Tumor volume	Volume of the tumor region (cm^3^)	0.47, [0.45, 0.52]
Radiomic phenotypes_ heterogeneity-mitigated	Radiomic phenotype generated from harmonized features	0.55, [0.52, 0.61]
Clinical covariates	PDL1, BMI, Smoking Status, ECOG	0.57, [0.53, 0.62]
Tumor volume + clinical covariates	Volume of the tumor region (cm^3^) + clinical covariates	0.58, [0.52, 0.60]
Radiomic phenotypes heterogeneity- mitigated + clinical covariates	Radiomic phenotypes (generated from harmonized features) + clinical covariates	0.63, [0.54, 0.64]

The multivariable PFS model with the radiomic phenotypes and clinical covariates of patients treated with monotherapy yielded a c-statistic of 0.55 (95% CI 0.51–0.61); the model with the radiomic phenotypes and clinical covariates of patients treated with combination therapy yielded a c-statistic of 0.60 (95% CI 0.52–0.62) (Table 6).

**Table 6:** Progression-free survival Cox proportional-hazards regression c-scores by line of therapy.

Model	Components	Fivefold cross-validated c-score, 95% CI
Radiomic phenotypes_ heterogeneity-mitigated_monotherapy + clinical covariates	Clinical covariates + radiomic phenotypes generated from harmonized features for patients treated with monotherapy. The heterogeneity in the image parameters has been mitigated by resampling and harmonization techniques	0.55, [0.51, 0.61]
Radiomic phenotypes_heterogeneity-mitigated_combination therapy + clinical covariates	Clinical covariates + radiomic phenotypes generated from harmonized features for patients treated with combination therapy. The heterogeneity in the image parameters has been mitigated by resampling and harmonization techniques	0.60, [0.52, 0.62]

### Biological significance of the radiomic phenotypes

**Figure 5:**
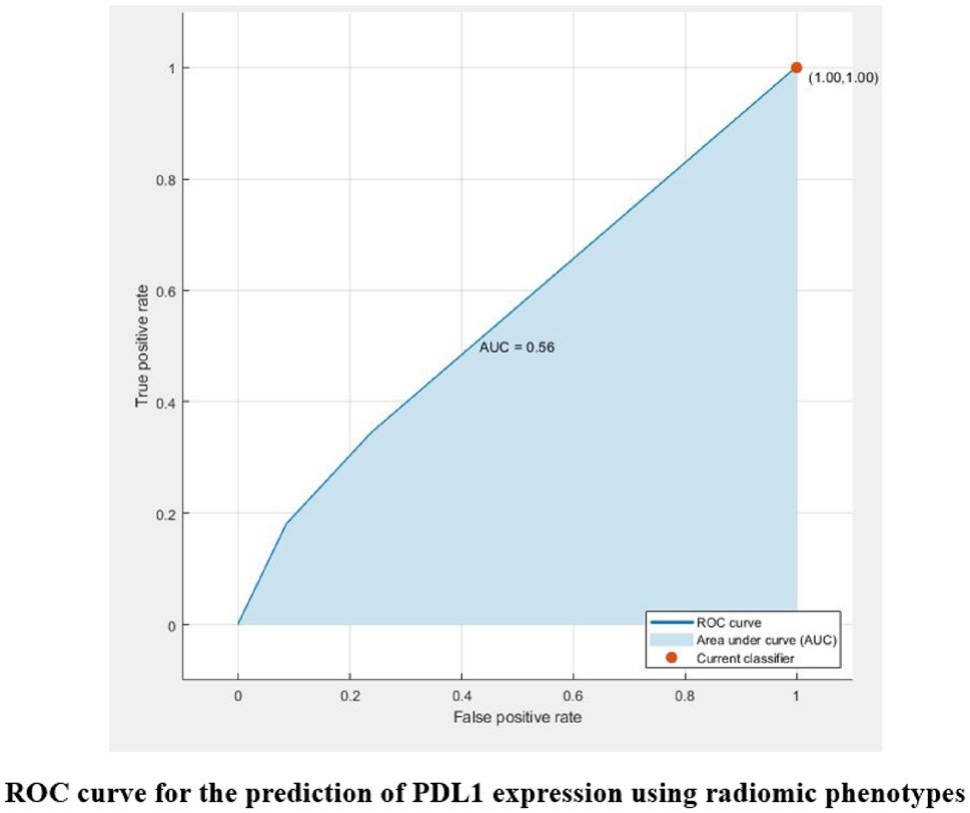
ROC curve for the prediction of PDL1 expression using radiomic phenotypes: radiomic phenotypes were used to predict PDL1 expression using a Random Forest classifier and obtained a fivefold cross-validated AUC of 0.56.

**Figure 6:**
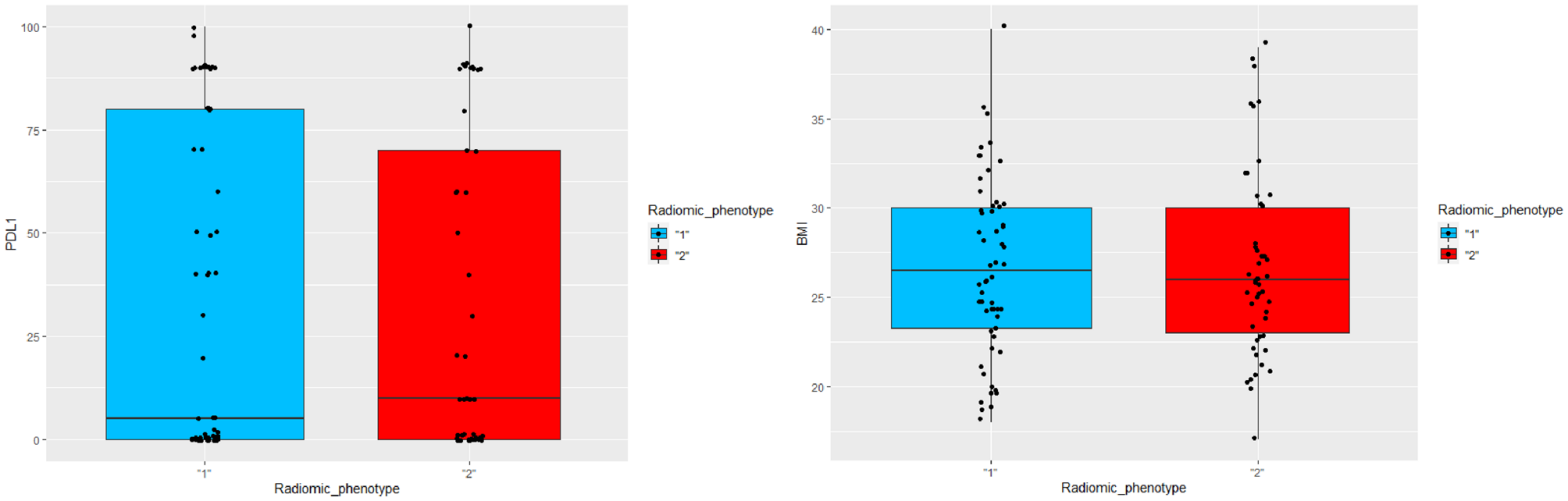
Distribution of PDL1 expression and BMI for radiomic phenotypes 1 and 2. The average value of PDL1 expression for patients belonging to phenotype 1 was 34.5% and those belonging to phenotype 2 was 33%. The average value of BMI for patients belonging to phenotype 1 was 26.7 and those belonging to phenotype 2 was 26.8. The differences in the mean values of PDL1 expression and BMI between patients belonging to phenotype 1 and phenotype 2 are not statistically significant.

Radiomic phenotypes were used to predict PDL1 expression using a Random Forest classifier and obtained a fivefold cross-validated AUC of 0.56 and a non-cross-validated AUC of 0.63 (Fig. 5). The average value of PDL1 expression for patients belonging to phenotype 1 was 34.5% and those belonging to phenotype 2 was 33%. The average value of BMI for patients belonging to phenotype 1 was 26.7 and those belonging to phenotype 2 was 26.8 (Fig. 6).

## Discussion

To our knowledge, no previous radiomics-based study has assessed the ability of radiomics to predict outcomes to first-line pembrolizumab in NSCLC. In addition, we address several areas of unmet need in the NSCLC immunotherapy literature including addressing the impact of heterogeneity of image acquisition and reconstruction parameters on the information extracted from the radiomic features and prediction modelling using individual radiomic features. In addition, we defined two radiomic tumor phenotypes which differ in terms of size and tumor.

**Figure 7:**
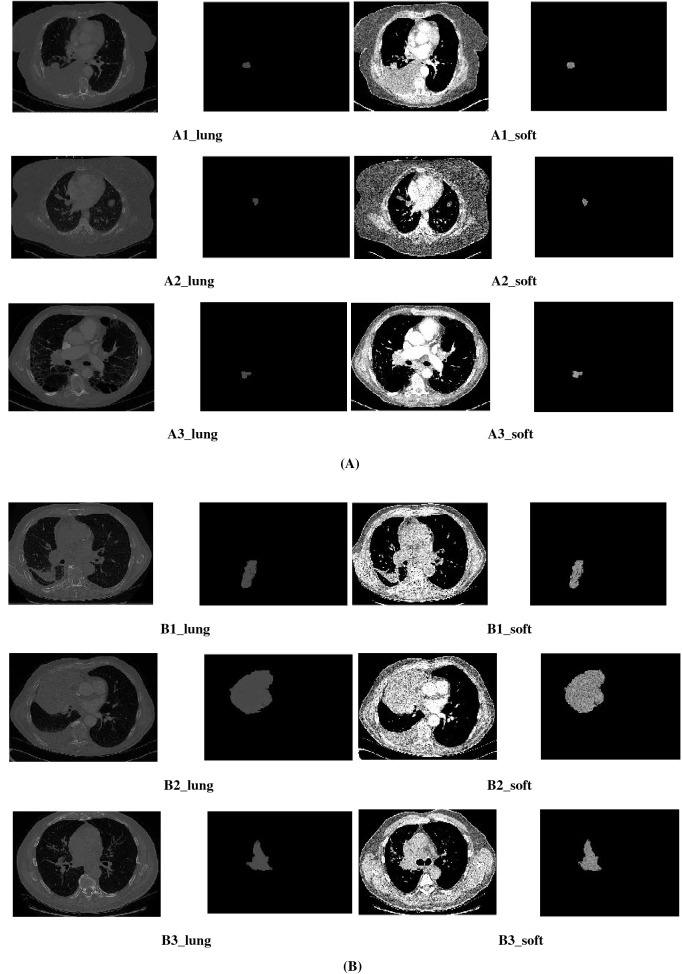
(**A**) Representative tumors belonging to phenotype 1- A_lung and A_soft represent the lung and soft tissue windows respectively. (**B**) Representative tumors belonging to phenotype 2- B_lung and B_soft represent the lung and soft tissue windows respectively.

The tumors in our sample demonstrate differences in terms of smoothness of the tumor surface boundaries between the two radiomic phenotypes. They also demonstrate differences in terms of size, with tumors belonging to phenotype 2 apparently bigger than tumors belonging to phenotype 1 and having potentially more irregular borders, but otherwise visually appearing similar morphologically (Fig. 7A and B). For example, the average tumor volume of the patients belonging to phenotype 1 is 40.2 cm^3^ and the average tumor volume of the patients belonging to phenotype 2 is 118.7 cm^3^. The tumor volumes of the representative tumors shown in Fig. 7A belonging to phenotype 1 are 7.6 cm^3^ (A1), 5.1 cm^3^ (A2) and 5.2 cm^3^. The tumor volumes of the representative tumors shown in Fig. 7B belonging to phenotype 2 are 85.3 cm^3^ (B1), 736.6 cm^3^ (B2) and 150.8 cm^3^ (B3). This observation suggests that while there may not be additional differences that are visually appreciable besides tumor size, the radiomics analysis may be able to capture patterns relevant to response to therapy and prognostic factors that may not be appreciable by the human eye such as internal tumor regions of tumor hypoxia in larger tumors. In addition, larger tumor**s** are known to have a worse prognosis with each increase in cm in diameter corresponding to a higher t-stage in NSCLC staging systems.

Our study demonstrates that use of a radiomic feature harmonization strategy (ComBat) to control the heterogeneity introduced by voxel spacing and image acquisition parameters improves the prognostic performance of the radiomic phenotypes. The phenotypes based on the features derived from images with heterogeneity in parameters mitigated using resampling and harmonization techniques, when combined with the clinical covariates, gave a c-statistic of 0.63, [0.54, 0.64] and a *statistically significant separation* (log-rank test *p* value = 0.00022) between the patients at low risk from those at high risk from therapy. In comparison, the volume of the tumor regions, when combined with the clinical covariates, could only give a c-statistic of 0.58, [0.52, 0.60]. A multivariable model built from the original features without ComBat harmonization yielded a c-statistic of 0.51 (95% CI 0.49–0.54) and could not produce a statistically significant separation (log-rank test *p* value = 0.16) between patients above versus below the median prognostic score. This suggests that radiomic features derived from those images where the heterogeneity in imaging parameters has been *mitigated* using resampling and harmonization techniques result in phenotypes with improved signature signal and better prognostic performance. Thus, we conclude that our imaging biomarker can be combined with PDL1 and other established clinical markers to enhance therapy response prediction. The multi-variable prognostic model built by combining our imaging biomarker with clinical markers gives a better prognostic performance compared to the model built by combining clinical markers with tumor volume, another important prognostic marker explored in previous studies^34,35^. Larger studies are warranted to fully validate these findings.

The phenotypes based on the features of patients treated with PEMBRO monotherapy, when combined with the clinical covariates, gave a c-statistic of 0.55, [0.51, 0.61], and could not produce a statistically significant separation (log-rank test *p* value = 0.32) between the low risk and high risk patients. The phenotypes based on features of patients treated with a combination of PEMBRO and chemotherapy, when combined with the clinical covariates, gave a c-statistic of 0.60, [0.52, 0.62], and gave a statistically significant separation (log-rank test *p* value = 0.0095) between the patients at low risk from those at high risk from therapy. This indicates that the model can predict progression-free survival for patients treated with combination therapy better than for those treated with monotherapy.

There are important limitations to our study. First, our study sample is relatively small. As a proof of concept, it is important to validate our findings with larger cohorts. Also, since most of the patients had adenocarcinoma and the patients with squamous carcinoma were only a small subset of our patient population. Thus, we did not separate patients based on their histology in our analysis. Further, we have performed manual segmentation of the tumor regions. There are studies that indicate the effect of inter-reader variability on tumor segmentation and radiomic feature extraction^36^. However, some recent studies suggest that such variability may not necessarily affect the robustness of all radiomic features^37^. A recent preliminary study showed that radiomic features extracted from segmentations obtained by different human observers tend to be highly correlated and have similar predictive value^38^. Our future work should evaluate the effect of inter-reader segmentation variability on radiomic features and explore fully automated algorithms.

## Conclusion

In this study, we developed a novel radiomic biomarker that integrates with PDL1 expression, ECOG status, BMI, and smoking status data to enhance the ability to predict progression-free survival in patients with stage 4 NSCLC, treated with first line PEMBRO monotherapy or combination therapy. We show that mitigation of the heterogeneity introduced by voxel spacing and image acquisition parameters improves the prognostic performance of the radiomic phenotypes. Our future work will explore the reproducibility of our results in a larger cohort of advanced NSCLC patients treated with first-line immunotherapy.

## Supplementary Information

Supplementary Information.

## Abbreviations

PD1: Programmed cell death protein 1
PDL1: Programmed death-ligand 1
PEMBRO: Pembrolizumab
NSCLC: Non-small cell lung cancer
OS: Overall survival
PFS: Progression-free survival
ROI: Region of interest
ECOG: Eastern Cooperative Oncology Group
BMI: Body mass index
