# Supplementary material for: Development of a robust radiomic biomarker of progression-free survival in advanced non-small cell lung cancer patients treated with first-line immunotherapy
Source: Sci Rep. 2022 Jun 15;12:9993. doi: 10.1038/s41598-022-14160-7 (PMC9200843; doi:10.1038/s41598-022-14160-7)
Supplement: Supplementary file 1 — Supplementary Information. [file 41598_2022_14160_MOESM1_ESM.docx]

**Development of a robust radiomic biomarker of progression-free survival in advanced non-small cell lung cancer patients treated with first-line immunotherapy**

Apurva Singh^1,2^, Hannah Horng ^2^, Leonid Roshkovan^1^, Joanna K. Weeks ^1^, Michelle Hershman ^1^, Peter Noël^1^, José Marcio Luna^1^, Eric A. Cohen^1^, Lauren Pantalone^1^, Russell T. Shinohara^3^, Joshua M. Bauml^4^, Jeffrey C. Thompson^4,5^, Charu Aggarwal^4^, Erica L. Carpenter^4^, Sharyn I. Katz^1^, Despina Kontos^1*^

^1^Department of Radiology, University of Pennsylvania, Philadelphia, PA, 19104

^2^Department of Bioengineering, University of Pennsylvania, Philadelphia, PA, 19104

^3^Department of Biostatistics, Epidemiology, and Informatics, University of Pennsylvania, Philadelphia, PA, 19104

^4^Department of Medicine, Division of Hematology-Oncology, University of Pennsylvania, Philadelphia, PA, 19104

^5^Department of Medicine, Pulmonary, Allergy and Critical Care Medicine, Thoracic Oncology Group, University of Pennsylvania, PA 19104

**Table S1: Details of therapy administered to the cohort.**

| **Type of therapy** | **Details of therapy** |
| --- | --- |
| Monotherapy | FLAT DOSE PEMBROLIZUMAB |
| Combination therapy | ABRAXANE/CARBOPLATIN/PEMBROLIZUMAB |
|  | CARBOPLATIN/PACLITAXEL/PEMBROLIZUMAB |
|  | MAINTENANCE PEMBROLIZUMAB and PEMETREXED after CARBOPLATIN |
|  | ABRAXANE/CARBOPLATIN+ PEMBROLIZUMAB |
|  | PEMBROLIZUMAB with PEMETREXED/CARBOPLATIN |

**Table S2: Percentage of features with significantly different distributions attributable to batch effects in the original features and after applying nested ComBat detected with AD test at a significance level of p < 0.05.**

|  | **Original** | **Nested ComBat** |
| --- | --- | --- |
| **Contrast Enhancement** | 18.6% | 13.6% |
| **Kernel Resolution** | 24.5% | 19.6% |


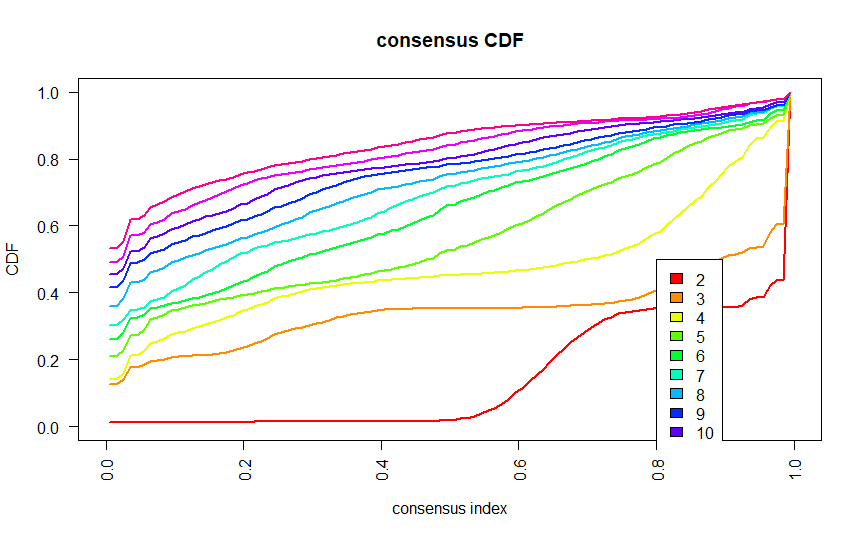

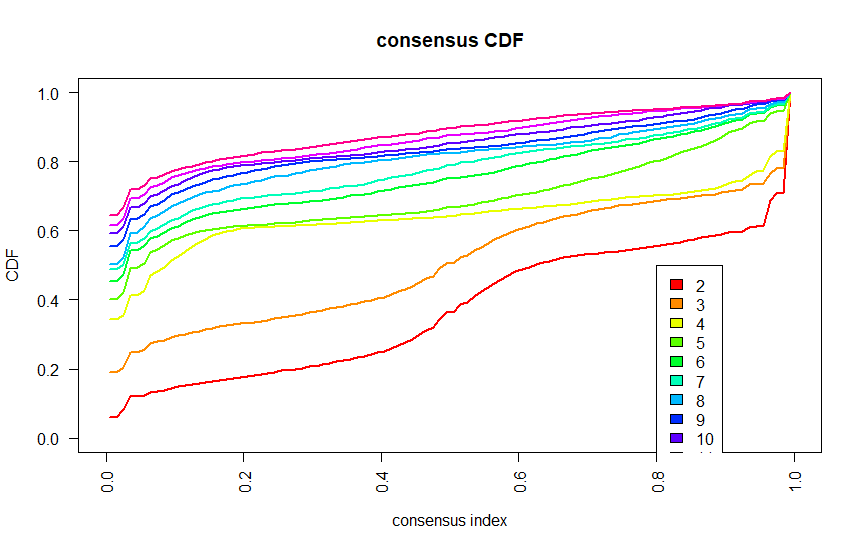


1. (b)

**Figure S1**: Consensus CDF from unsupervised hierarchical clustering for phenotype generation in (a) non-COMBAT harmonized features and (b) COMBAT harmonized features.


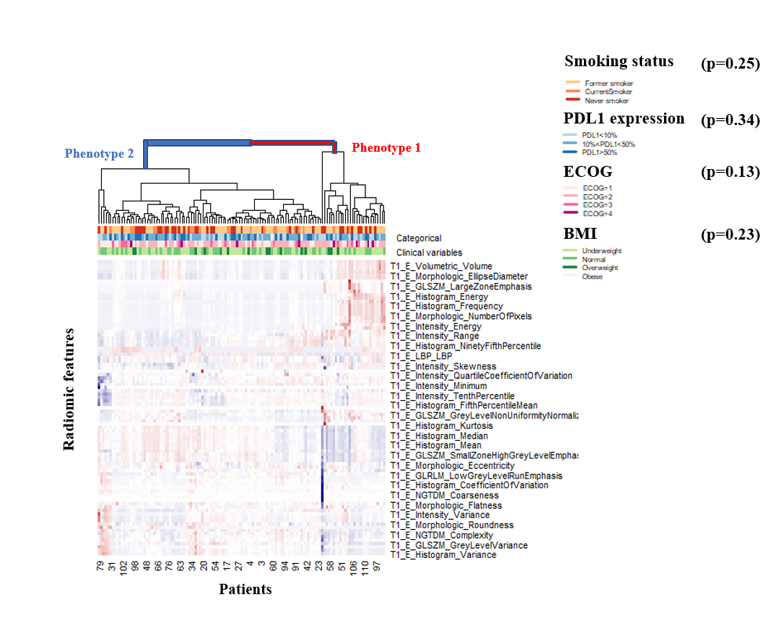


**Figure S2:** Heatmap of radiomic derived features. Unsupervised hierarchical clustering identifies two distinct, and statistically significant (p=0.02) tumor radiomic phenotypes. Association of these phenotypes with the clinical covariates is assessed by the Chi square test and the resultant p values are included in the figure. The p values in case of all the covariates is greater than 0.05. This shows that the radiomic phenotypes are not strongly associated with the clinical covariates.

**
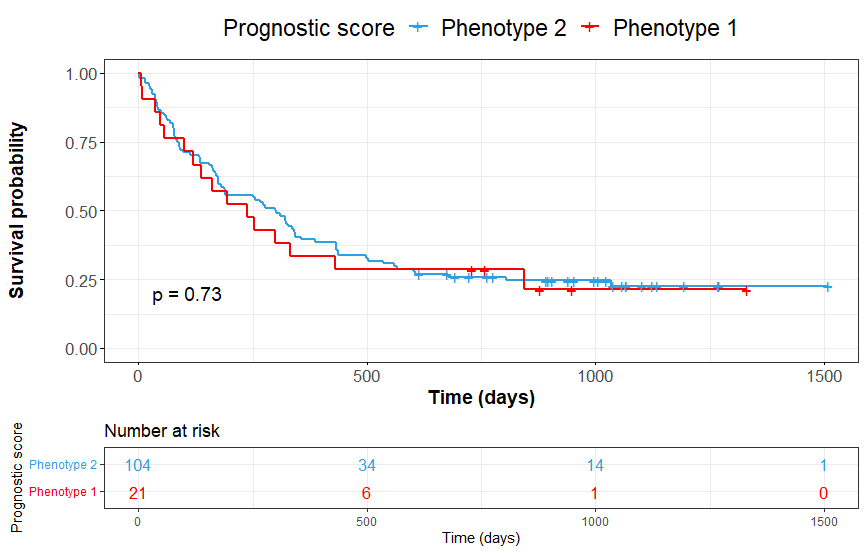
**

**Figure S3:** Survival analysis by radiomic phenotypes generated from non-harmonized radiomic features.

**
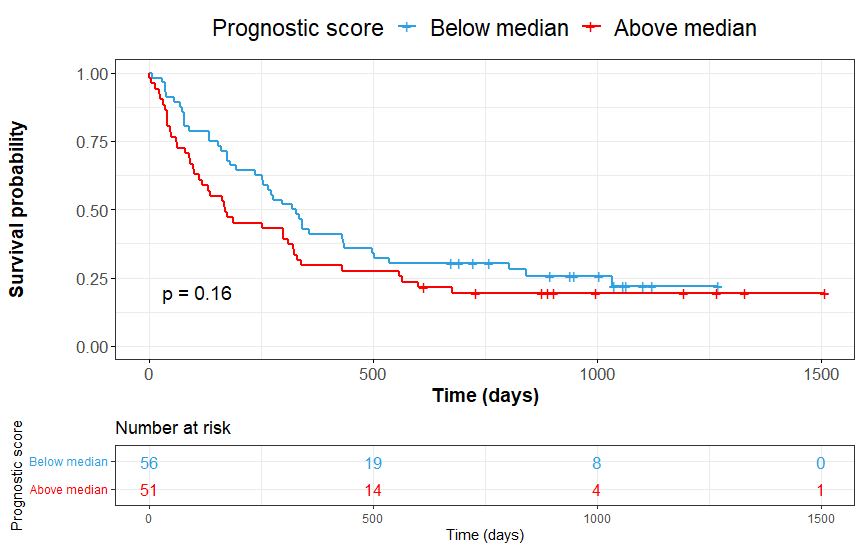
**

**Figure S4:** Survival analysis using the multivariable model built using clinical covariates and radiomic phenotypes generated from non-harmonized features.

**Table S3:** Progression-free survival Cox proportional-hazards regression analysis c-scores for a model fitted with phenotypes derived from non-harmonized radiomic features.

| **Model** | **Components** | **Five-fold cross-validated hazard ratio, 95% CI** |
| --- | --- | --- |
| **Radiomic phenotypes_non-harmonized+clinical features** | Radiomic phenotypes generated from non-harmonized features + clinical covariates | 0.51, [0.49,0.54] |

**Table S4**: A list of the radiomic features (102) extracted using CaPTk. The column headings indicate the family to which the list of features belongs to.

| **Intensity** | **Histogram** | **Volumetric** | **Morphologic** | **GLRLM** | **GLSZM** | **NGTDM** | **LBP** |
| --- | --- | --- | --- | --- | --- | --- | --- |
| Coefficient of Variation | Frequency | Pixels | Eccentricity | Grey Level Non- Uniformity Normalized | Grey Level Mean | Busyness | LBP |
| Energy | Coefficient of Variation | Volume | Ellipse Diameter | Grey Level Non-Uniformity | Grey Level Non- Uniformity | Coarseness |  |
| Inter Quartile Range | Energy |  | Elongation | Grey Level Variance | Grey Level Non-Uniformity Normalized | Complexity |  |
| Kurtosis | Entropy |  | Equivalent Spherical | High Grey Level Run Emphasis | Grey Level Variance | Contrast |  |
| Maximum | Fifth Percentile |  | Perimeter | Long Run Emphasis | High Grey Level Emphasis | Strength |  |
| Mean | Fifth Percentile Mean |  | Equivalent Spherical Radius | Long Run High Grey Level Emphasis | Large Zone Emphasis |  |  |
| Mean Absolute Deviation | Inter Quartile Range |  | Flatness | Long Run Low Grey Level Emphasis | Large Zone High Grey Level Emphasis |  |  |
| Median | Kurtosis |  | Largest Component Size | Low Grey Level Run Emphasis | Large Zone Low Grey Level Emphasis |  |  |
| Median Absolute Deviation | Mean |  | Number of Pixels | Run Entropy | Low Grey Level Emphasis |  |  |
| Minimum | Mean Absolute Deviation |  | Perimeter | Run Length Non- Uniformity Normalized | Small Zone Emphasis |  |  |
| Mode | Median |  | Physical Size | Run Length Non- Uniformity | Small Zone High Grey Level Emphasis |  |  |
| Ninetieth Percentile | Median Absolute Deviation |  | Roundness | Run Length Variance | Small Zone Low Grey Level Emphasis |  |  |
| Quartile Coefficient of Variation | Mode |  |  | Run Percentage | Zone Percentage |  |  |
| Range | Ninetieth Percentile |  |  | Short Run Emphasis | Zone Size Entropy |  |  |
| Root Mean Square | Ninety Fifth Percentile |  |  | Short Run High Grey Level Emphasis | Zone Size Mean |  |  |
| Skewness | Ninety Fifth Percentile Mean |  |  | Short Run Low Grey Level Emphasis | Zone Size Non- Uniformity |  |  |
| Standard Deviation | Quartile Coefficient of Variation |  |  | Total Runs | Zone Size Non-Uniformity Normalized |  |  |
| Sum | Robust Mean Absolute Deviation |  |  |  | Zone Size Variance |  |  |
| Tenth Percentile | Root Mean Square |  |  |  |  |  |  |
| Variance | Seventy Fifth Percentile |  |  |  |  |  |  |
|  | Skewness |  |  |  |  |  |  |
|  | Standard Deviation |  |  |  |  |  |  |
|  | Sum |  |  |  |  |  |  |
|  | Tenth Percentile |  |  |  |  |  |  |
|  | Twenty Fifth Percentile |  |  |  |  |  |  |
|  | Uniformity |  |  |  |  |  |  |
|  | Variance |  |  |  |  |  |  |

**Table S5**: Mathematical formulae describing the features.

| **Feature family** | **Description** |
| --- | --- |
| Intensity Features (First-Order Statistics) | - Minimum Intensity = Min (I_k_). where I_k_ is the intensity of pixel or voxel at index k. - Maximum Intensity = Max (I_k_). where I_k_ is the intensity of pixel or voxel at index k. - Mean= Σ(X_i_) where N is the number of   N  voxels/pixels.     - Standard Deviation = √(X-μ)^2^ where μ is the   N  mean of the data.   - Variance = (X-μ)^2^ where μ is the mean intensity.   N   - Skewness = Σ _i=1 to N (_X_i_-$\overline{X}$)^3^/N   s^3^  where $\overline{X}$ is the mean, s is the standard deviation and N is the number of pixels/voxels.   - Kurtosis = Σ _i=1 to N_ (X_i_-$\overline{X}$)^4^/N   s^4^  where  $\overline{X}$ is the mean, s is the standard deviation and N is the number of pixels/voxels. |
| Histogram-based features | - Uses number of bins as input and the number of pixels in each bin would be the output.   All features in this family are extracted from the discretized intensities. |
| Volumetric | - Volume/Area (depending on image dimension) and number of voxels/pixels in the ROI. |
| Morphologic | - Elongation = √(i_2_/i_1_) where in are the second moments of particle around its principal axes. - Perimeter = 2πr where r is the radius of the circle enclosing the shape. - Roundness = A_s_/A_c_= (Area of a shape)/ (Area of circle) where circle has the same perimeter. - Eccentricity = √[1- ((a+b)/c^2^)] where c is the longest semi-principal axis of an ellipsoid fitted on an ROI, and a and b are the 2nd and 3rd longest semi-principal axes of the ellipsoid. |
| GLRLM | For a given image, a run-length matrix P(i;j) is defined as the number of runs with pixels of gray level i and run length j.   - Short Run Emphasis (SRE) = 1 Σ_i,jtoN_ p(i,j)   n_r_ j^2^   - Long Run Emphasis (LRE) = 1 Σ_jtoN_ p(i,j).j^2^   n_r_   - Grey Level Non-uniformity (GLN) =   1 Σ_itoM_(Σ_jtoN_ p(i,j))^2^  n_r_   - Run Length Non-uniformity (RLN) =    1 Σ_i to M_ p_g_(i)  n_r_  i^2^     - High Grey-Level Run Emphasis   (HGRE)= 1 Σ_i to M_ p_g_(i). i^2^  n_r_   - Short Run Low Grey-Level Emphasis   (SRLGE)= 1 Σ_i to M_ Σ_j to N_ p(i,j)  n_r_ i^2^.j^2^   - Short Run High Grey-Level Emphasis   (SRHGE) = 1 Σ_i to M_ Σ_j to N_ p(i,j).i^2^  n_r_  j^2^   - Long Run Low Grey-Level Emphasis   (LRLGE) = 1 Σ_i to M_ Σ_j to N_ p(i,j).j^2^  n_r_  i^2^   - Long Run High Grey-Level Emphasis   (LRHGE) = 1 Σ_i to M_ Σ_j to N_ p(i,j).i^2^.j^2^  n_r_  All features are estimated within the ROI in an image, considering 26-connected neighbouring voxels in the 3D volume. |
| GLSZM | For a given image, a run-length matrix P(i;j) is defined as the number of runs with pixels of gray level i and run length j.   - Small Zone Emphasis (SZE) = 1 Σ_i,jtoN_ p(i,j)   n_r_ j^2^   - Large Zone Emphasis (LZE) = 1 Σ_jtoN_ p(i,j).j^2^   n_r_   - Gray-Level Non-uniformity (GLN) =   1 Σ_itoM_ (Σ_jtoN_ p(i,j))^2^  n_r_   - Zone-Size Non-uniformity (ZSN) =   1 Σ_jtoN_ (Σ_itoM_ p(i,j))^2^  n_r_   - Zone Percentage (ZP) = n_r_/n_p_ where n_r_ is the total number of runs and n_p_ is the number of pixels in the image. - Low Grey-Level Zone Emphasis (LGZE)=   1 Σ_i to M_ p_g_(i)  n_r_  i^2^   - High Grey-Level Zone Emphasis (HGZE)=   1 Σ_i to M_ p(i,j).i^2^  n_r_   - Short Zone Low Grey-Level Emphasis (SZLGE)=   1 Σ_i to M_ Σ_j to N_ p(i,j)  n_r_ i^2^. j^2^   - Short Zone High Grey-Level Emphasis (SZLGE) =   1 Σ_i to M_ Σ_j to N_ p(i,j).i^2^  n_r_  j^2^   - Long Zone Low Grey-Level Emphasis (LZLGE) =   1 Σ_i to M_ Σ_j to N_ p(i,j).j^2^  n_r_  i^2^   - Long Zone High Grey-Level Emphasis (LZHGE) =   1 Σ_i to M_ Σ_j to N_ p(i,j).i^2^.j^2^  n_r_  All features are estimated within the ROI in an image, considering 26-connected neighbouring voxels in the 3D volume. |
| NGTDM | - Coarseness = [ε +Σ _i=0toGk_ p_i_ s(i)] - Contrast =   [ 1 Σ_itoGk_ Σ_jtoGk_ pipj(i-j)^2^][1 Σ_itoGk_ s(i)]  Ns(Ns-1) n^2^   - Busyness =  [Σ_itoGk_ p_i_s(i)]   [Σ_itoGk_ Σ_itoGk_ ip_i_-jp_j_]   - Complexity = Σ_itoGk_ Σ_jtoGk_[(│i−j│)] [p_i_ s(i)+p_j_ s(j)]   (n^2^(p_i_+p_j_))   - Strength =    [Σ_itoGk_Σ_jtoGk_(p_i_+p_j_)(i-j)^2^]/[ε+Σ_itoGk_ s(i)]  Where pi is the probability of occurrence of a voxel of intensity i and s(i) represents the NGTDM value of intensity i calculated as: ∑│i−Ai│. Ai indicates the average intensity of the surrounding voxels without including the central voxel. |
| LBP | The pixel-wise LBP codes are computed using N number of neighbours on a circle of radius R around each pixel and using a rotation invariant implementation. The output value corresponds to the mean of the LBP map. |
